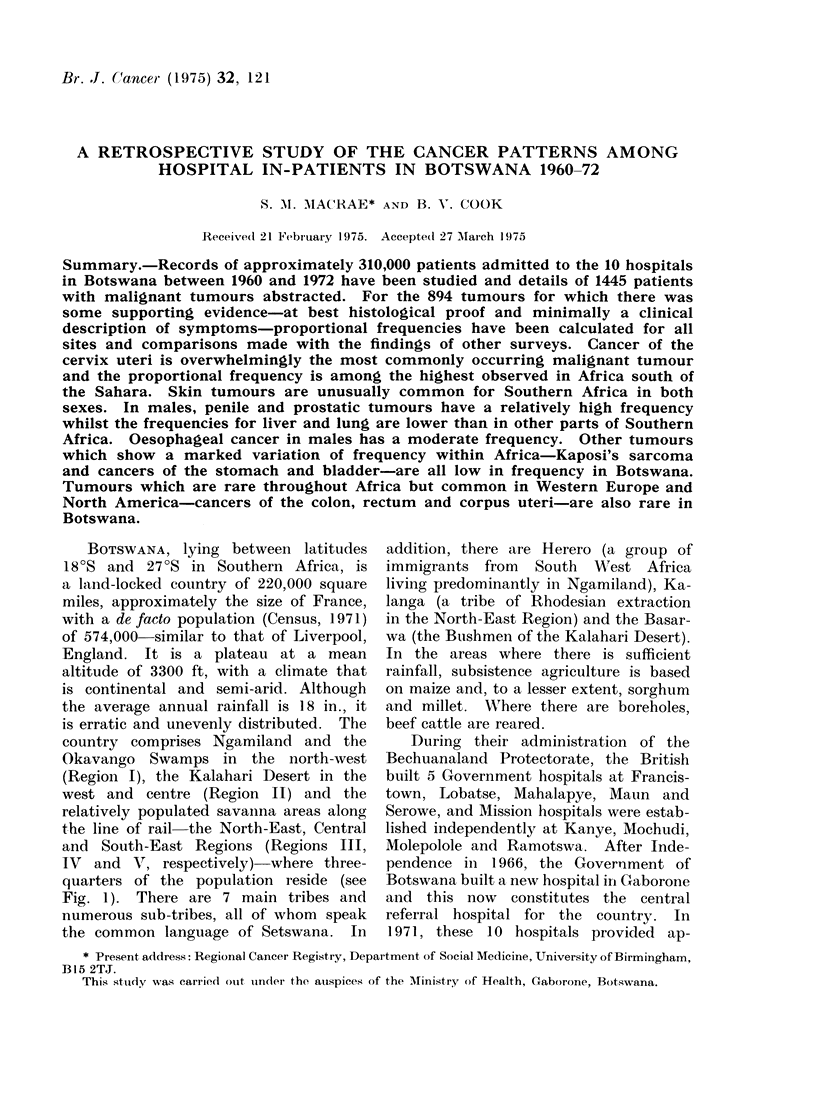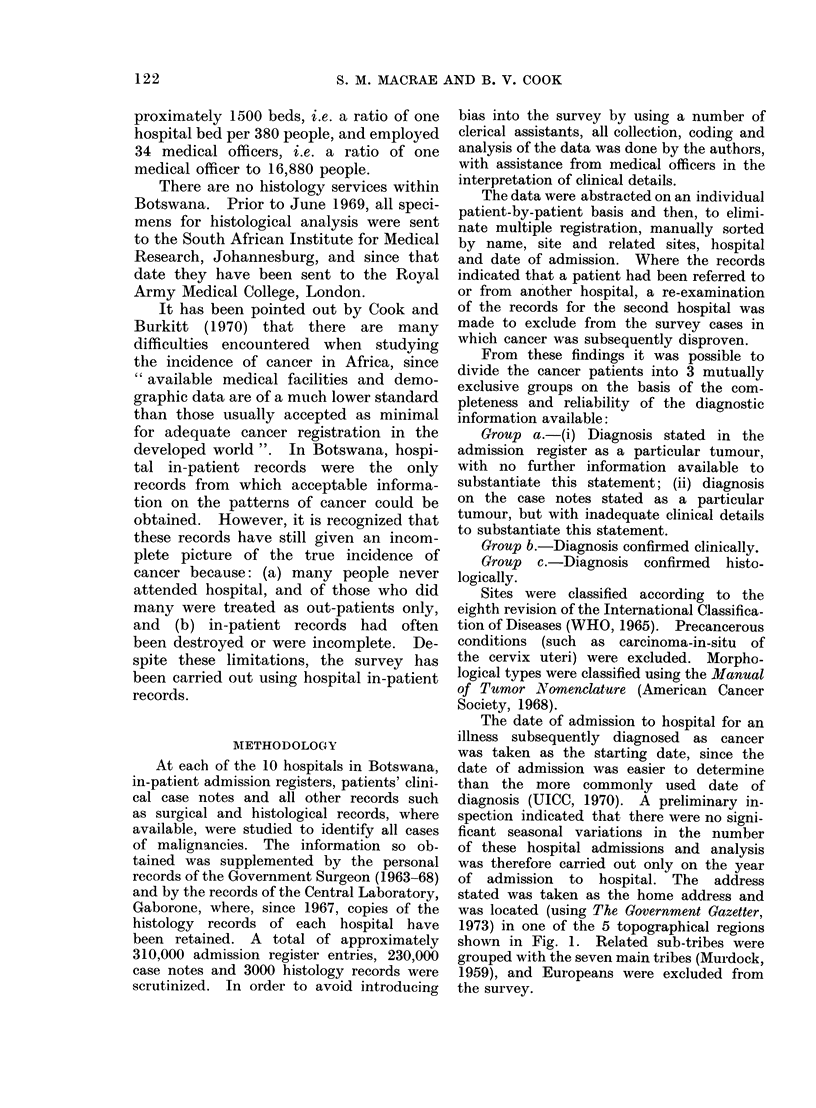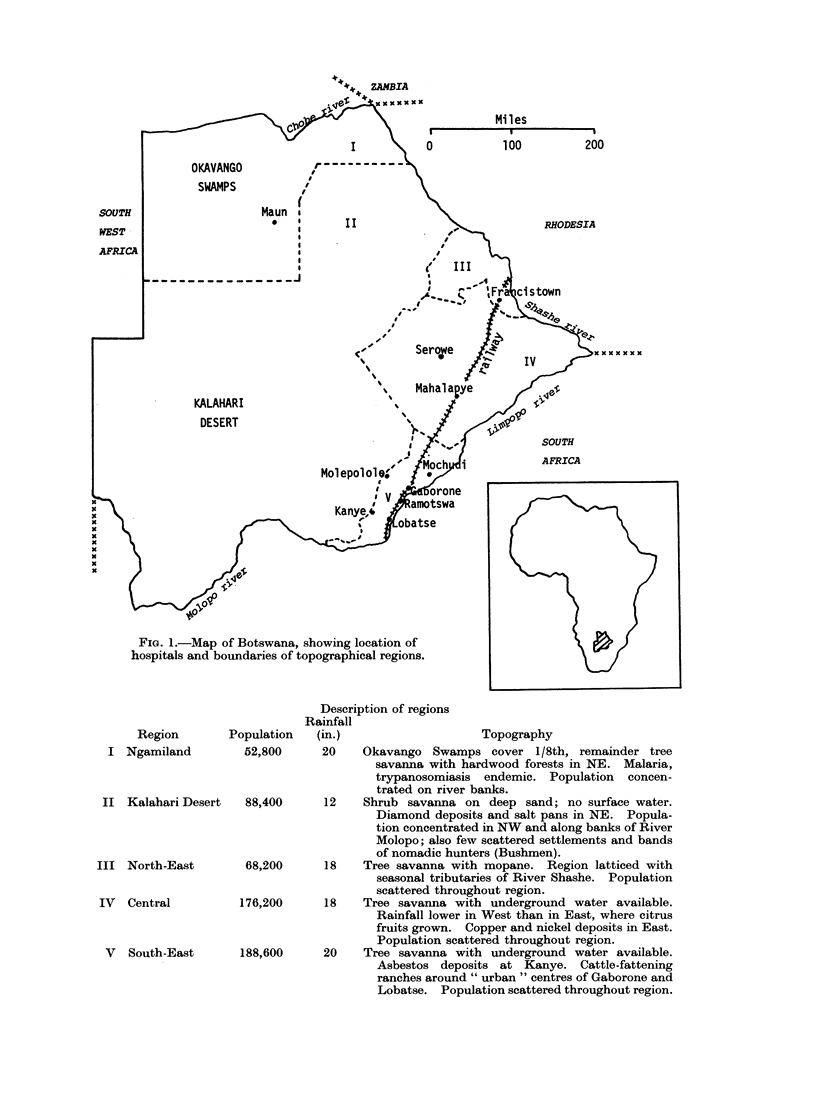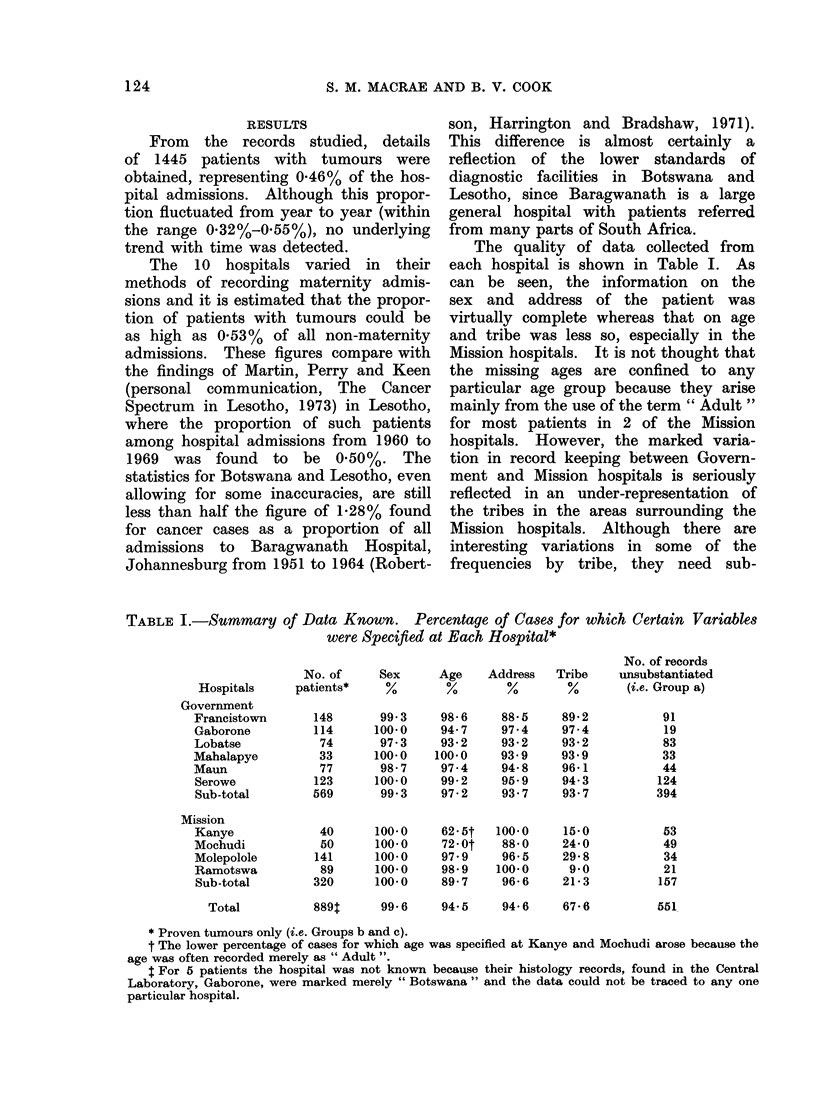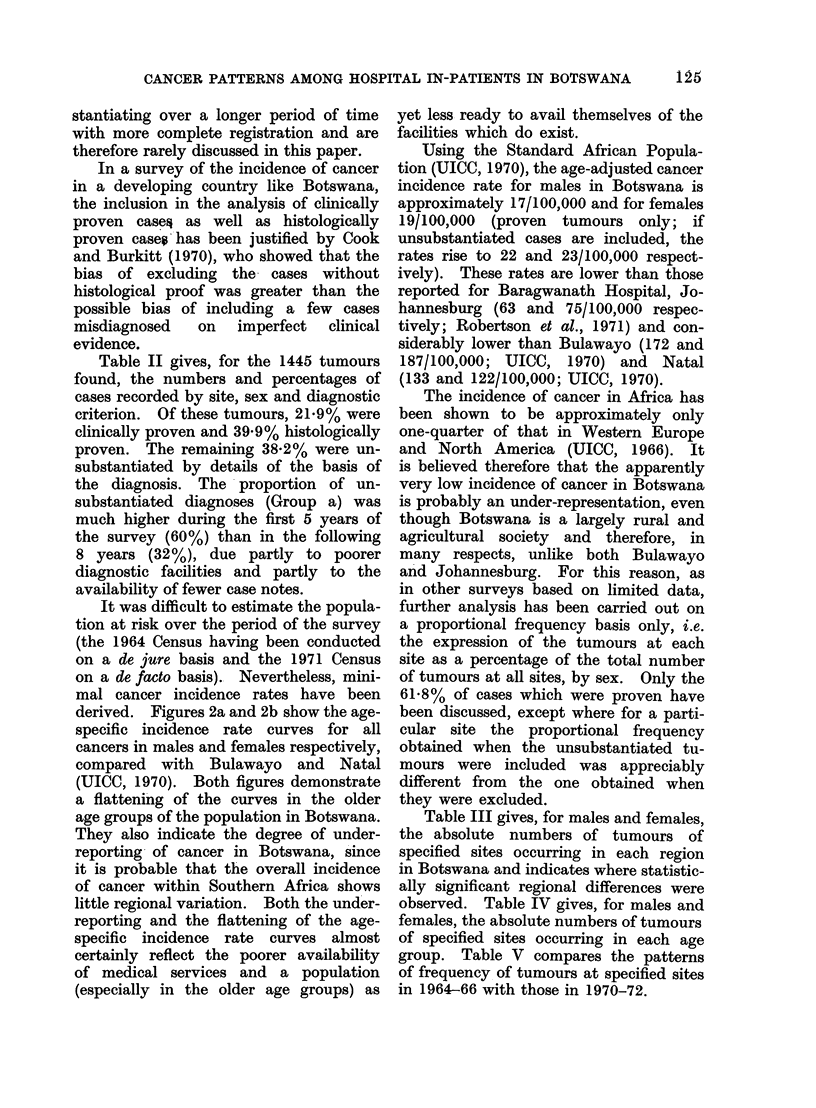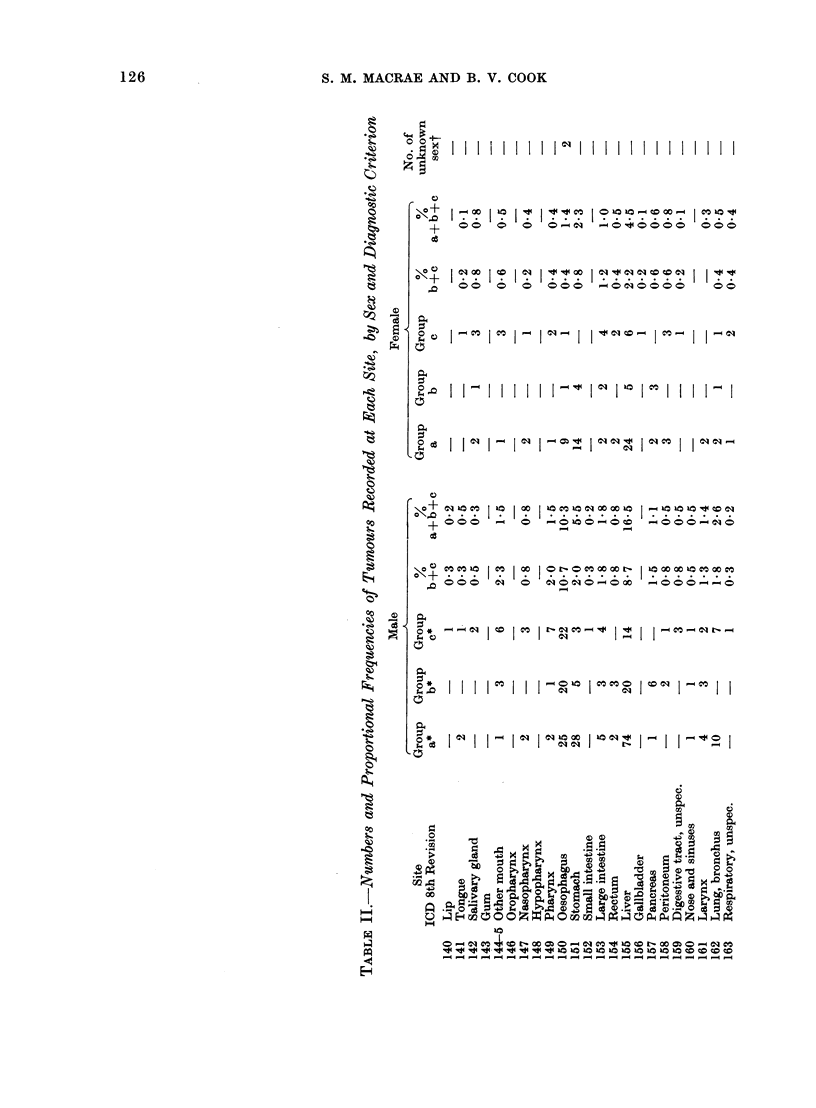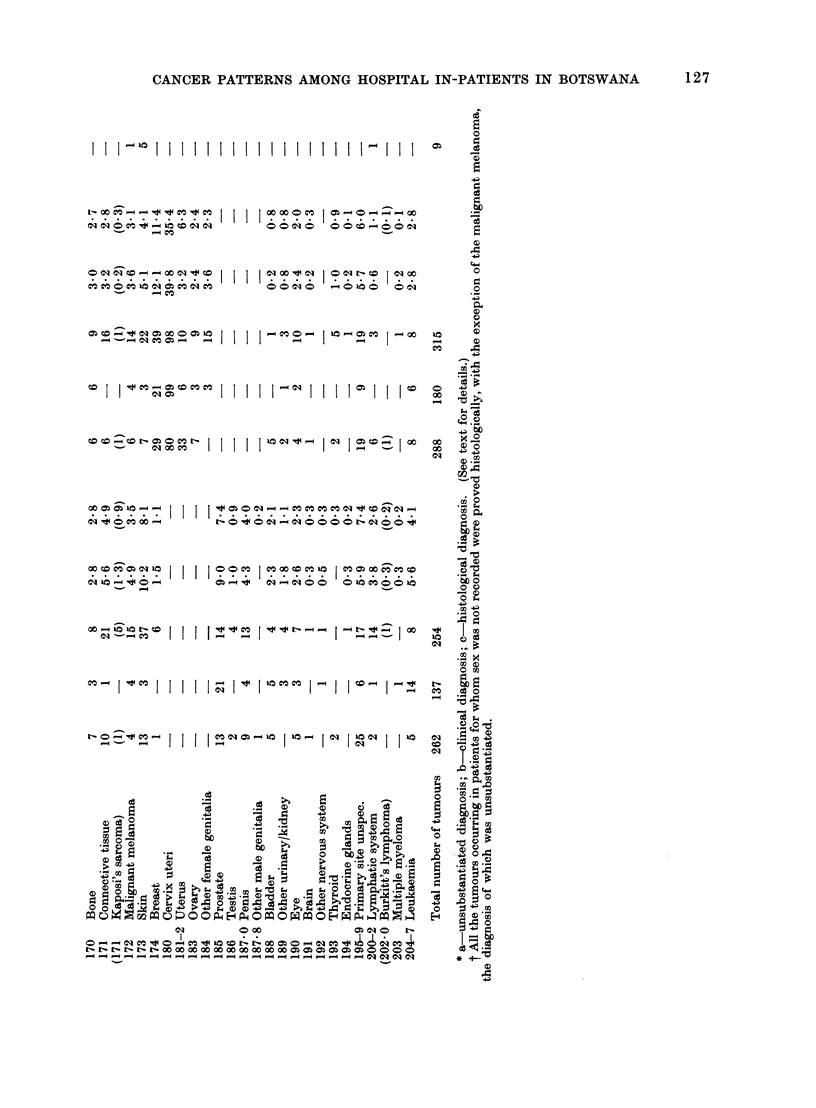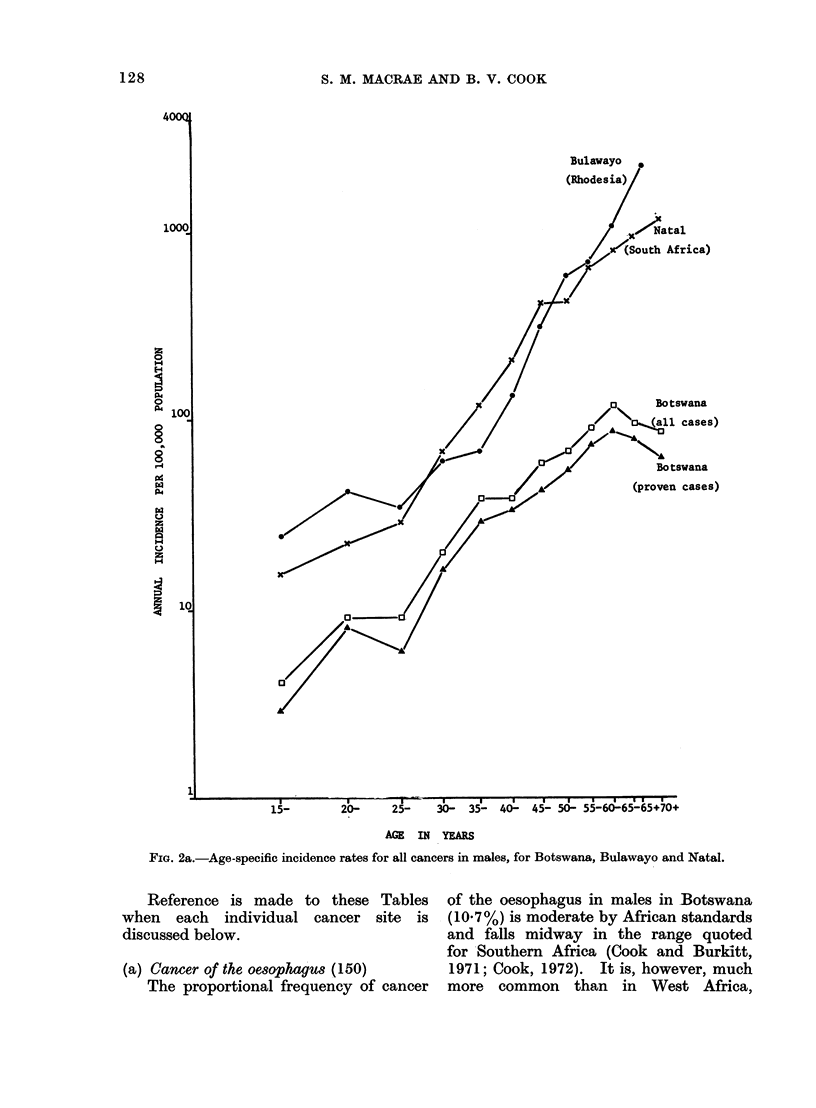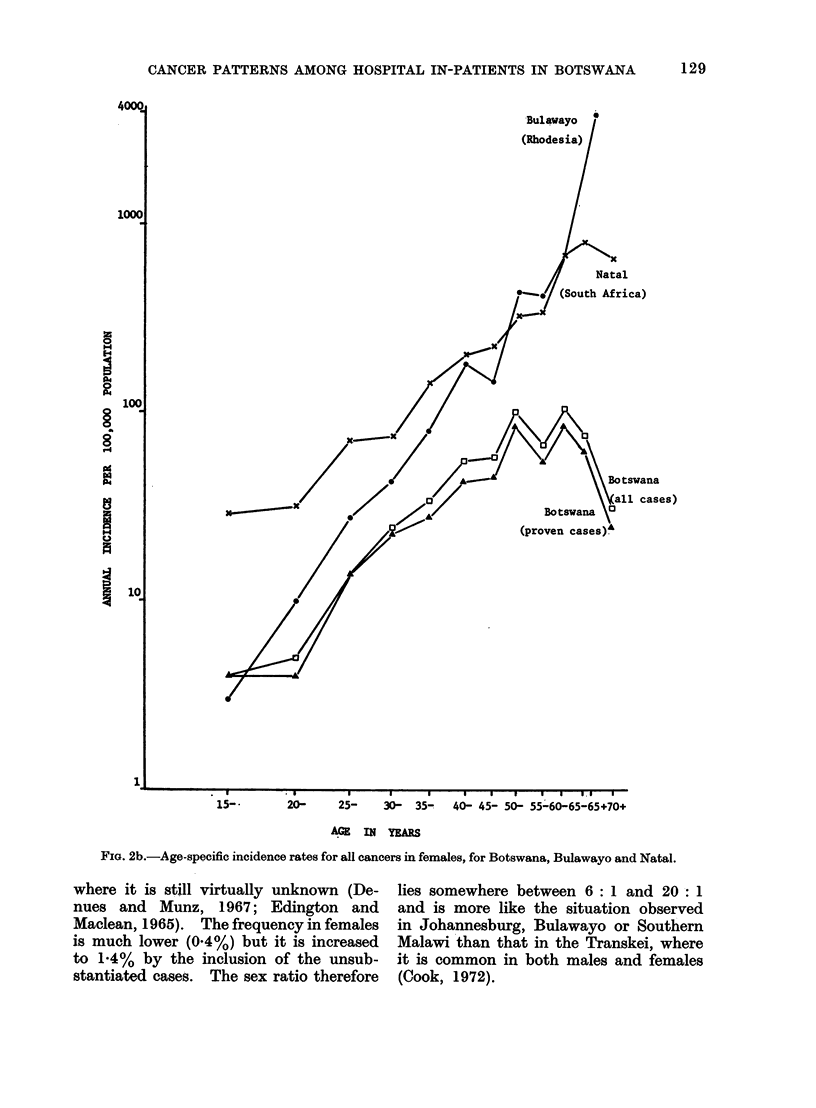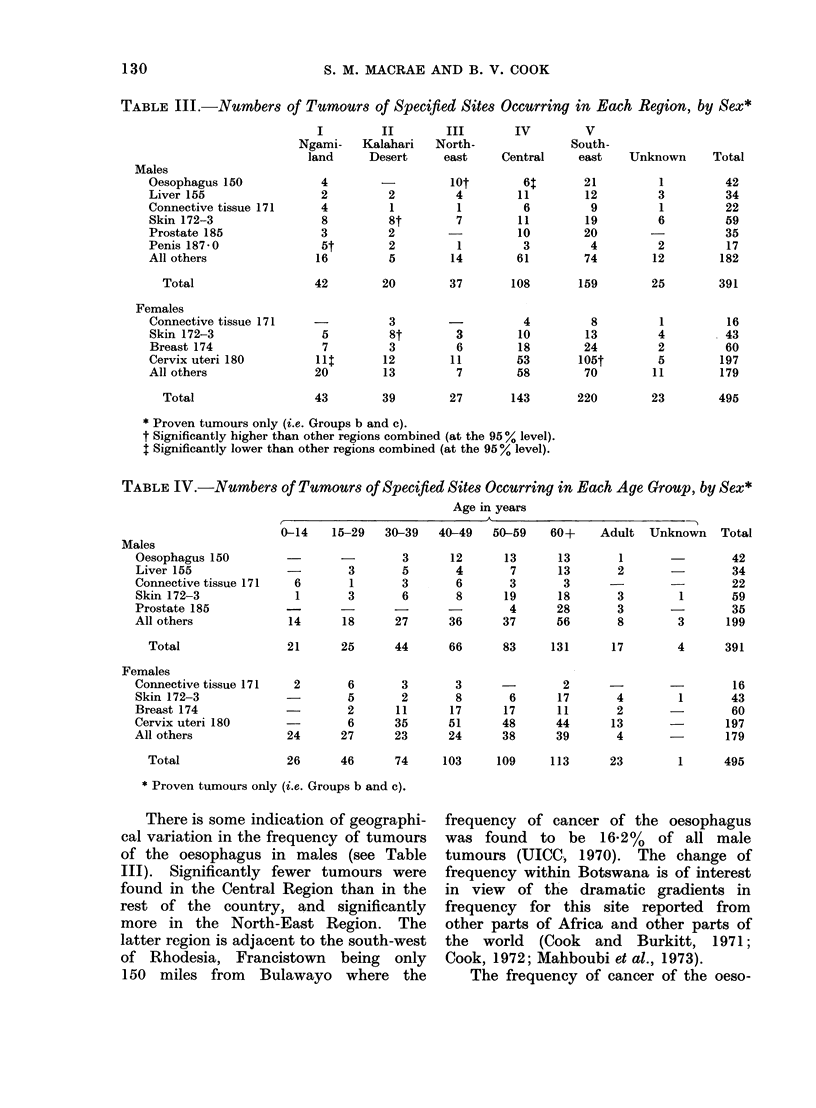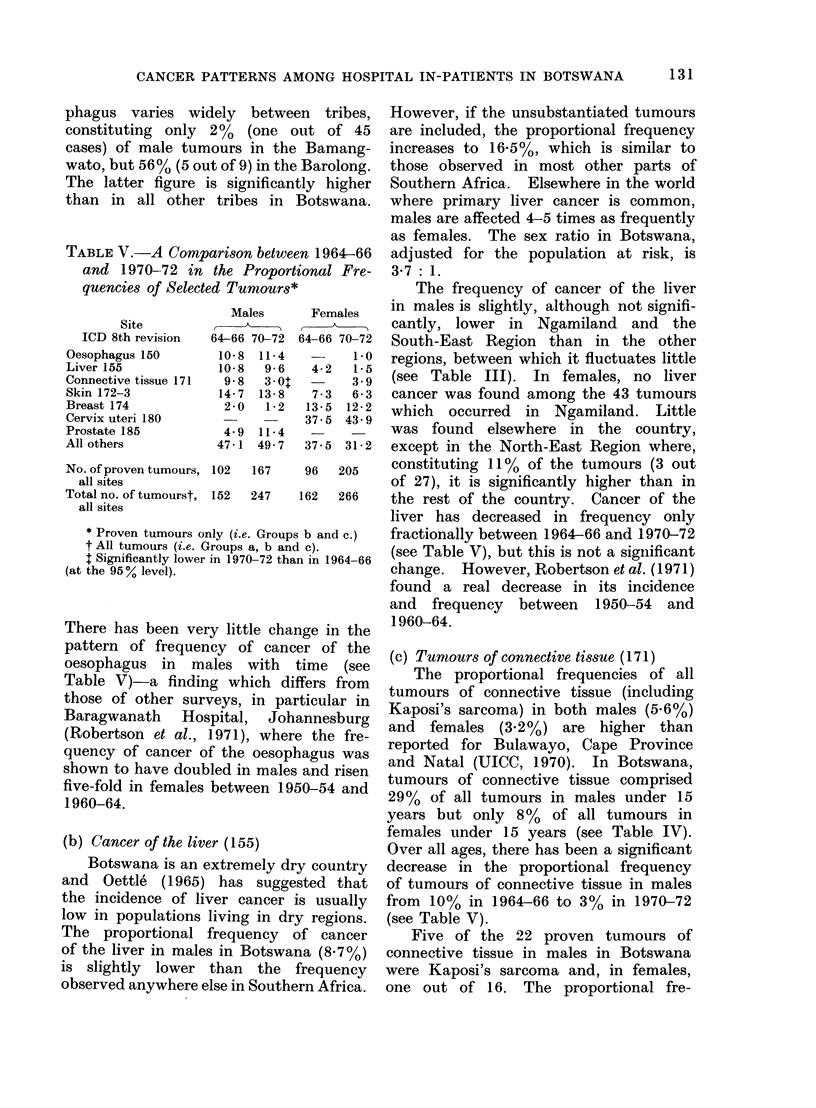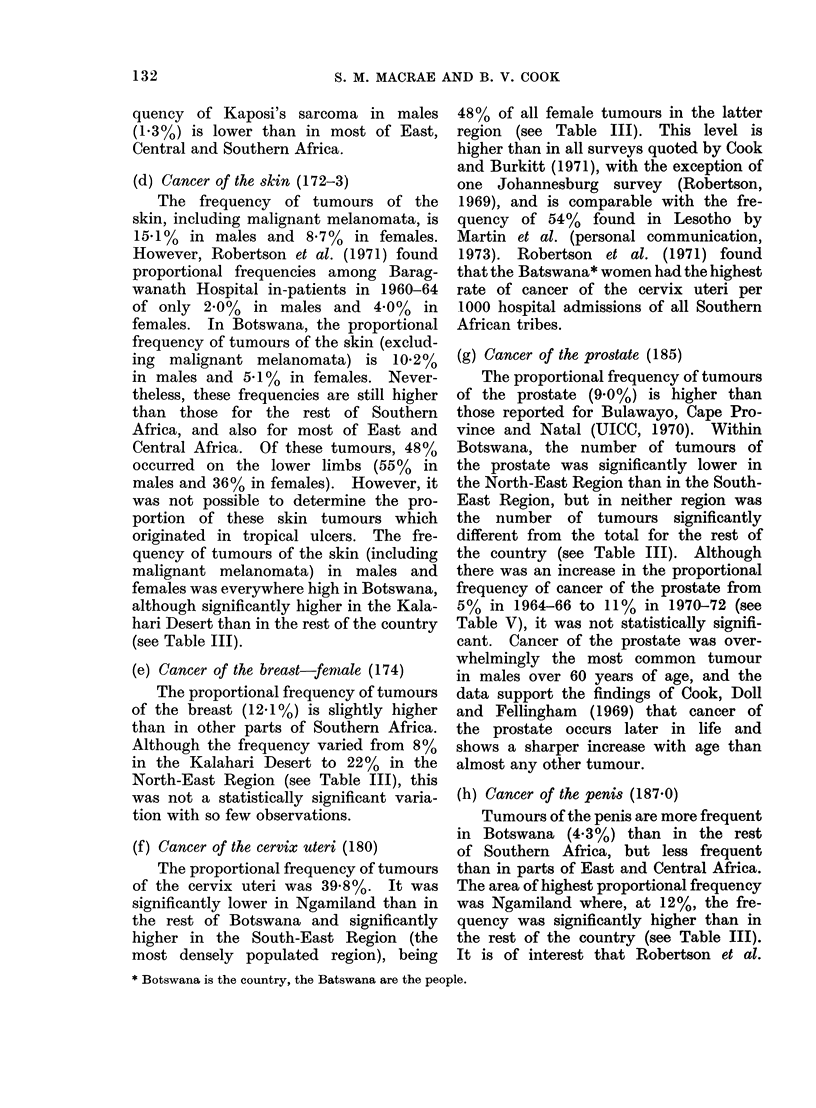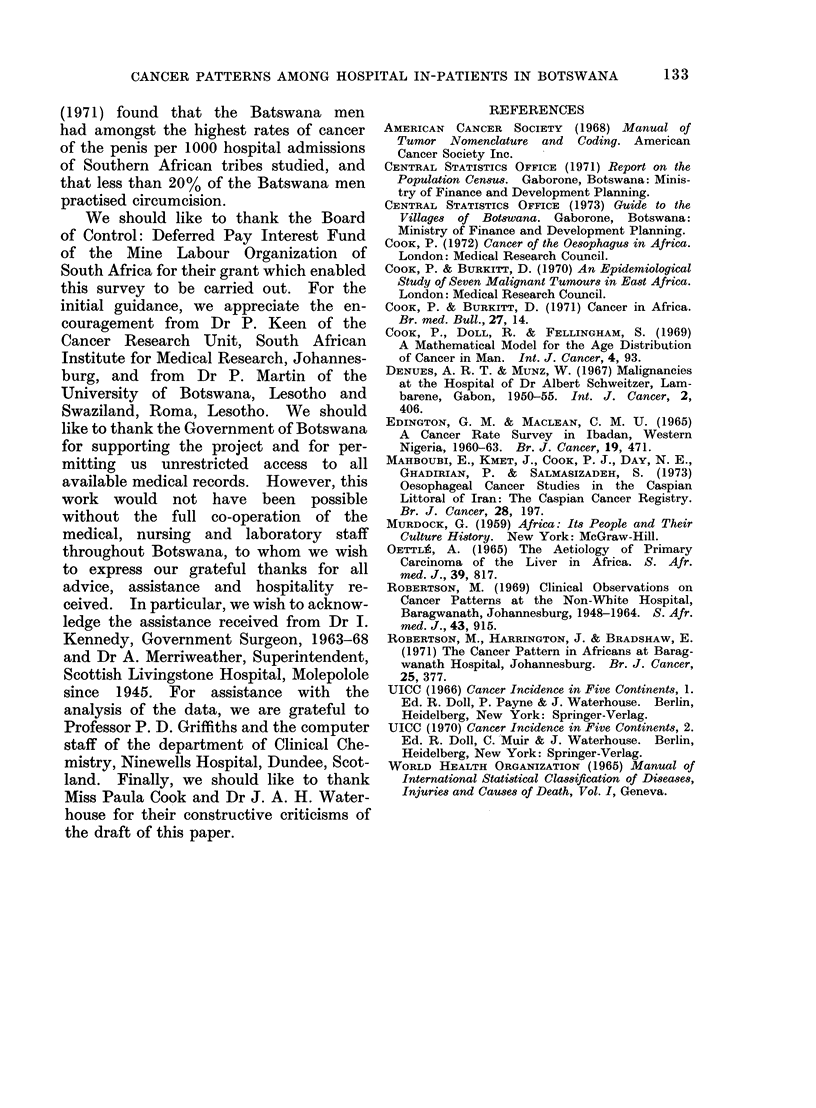# A retrospective study of the cancer patterns among hospital in-patients in Botswana 1960-72.

**DOI:** 10.1038/bjc.1975.138

**Published:** 1975-07

**Authors:** S. M. Macrae, B. V. Cook

## Abstract

Records of approximately 310,000 patients admitted to the 10 hospitals in Botswana between 1960 and 1972 have been studied and details of 1445 patients with malignant tumours abstracted. For the 894 tumours for which there was some supporting evidence--at best histological proof and minimally a clinical description of symptoms--proportional frequencies have been calculated for all sites and comparison made with the findings of other surveys. Cancer of the cervix uteri is overwhelmingly the most commonly occurring malignant tumour and the proportional frequency is among the highest observed in Africa south of the Sahara. Skin tumours are unusually common for Southern Africa in both sexes. In males, penile and prostatic tumours have a relatively high frequency whilst the frequencies for liver and lung are lower than in other parts of Southern Africa. Oesophageal cancer in males has a moderate frequency. Other tumours which show a marked variation of frequency within Africa--Kaposi's sarcoma and cancers of the stomach and bladder--are all low in frequency in Botswana. Tumours which are rare throughout Africa but common in Western Europe and North America--cancers of the colon, rectum and corpus uteri--are also rare in Botswana.


*Br. J. Cancer* (1975) **32**, 121

### A RETROSPECTIVE STUDY OF THE CANCER PATTERNS AMONG HOSPITAL IN-PATIENTS IN BOTSWANA 1960-72

S. M. MACRAE* AND B. V. COOK

Received 21 February 1975. Accepted 27 March 1975

**Summary.—Records of approximately 310,000 patients admitted to the 10 hospitals in Botswana between 1960 and 1972 have been studied and details of 1445 patients with malignant tumours abstracted. For the 894 tumours for which there was some supporting evidence—at best histological proof and minimally a clinical description of symptoms—proportional frequencies have been calculated for all sites and comparisons made with the findings of other surveys. Cancer of the cervix uteri is overwhelmingly the most commonly occurring malignant tumour and the proportional frequency is among the highest observed in Africa south of the Sahara. Skin tumours are unusually common for Southern Africa in both sexes. In males, penile and prostatic tumours have a relatively high frequency whilst the frequencies for liver and lung are lower than in other parts of Southern Africa. Oesophageal cancer in males has a moderate frequency. Other tumours which show a marked variation of frequency within Africa—Kaposi's sarcoma and cancers of the stomach and bladder—are all low in frequency in Botswana. Tumours which are rare throughout Africa but common in Western Europe and North America—cancers of the colon, rectum and corpus uteri—are also rare in Botswana.**

BOTSWANA, lying between latitudes 18°S and 27°S in Southern Africa, is a land-locked country of 220,000 square miles, approximately the size of France, with a *de facto* population (Census, 1971) of 574,000—similar to that of Liverpool, England. It is a plateau at a mean altitude of 3300 ft, with a climate that is continental and semi-arid. Although the average annual rainfall is 18 in., it is erratic and unevenly distributed. The country comprises Ngamiland and the Okavango Swamps in the north-west (Region I), the Kalahari Desert in the west and centre (Region II) and the relatively populated savanna areas along the line of rail—the North-East, Central and South-East Regions (Regions III, IV and V, respectively)—where three-quarters of the population reside (see Fig. 1). There are 7 main tribes and numerous sub-tribes, all of whom speak the common language of Setswana. In addition, there are Herero (a group of immigrants from South West Africa living predominantly in Ngamiland), Kalanga (a tribe of Rhodesian extraction in the North-East Region) and the Basarwa (the Bushmen of the Kalahari Desert). In the areas where there is sufficient rainfall, subsistence agriculture is based on maize and, to a lesser extent, sorghum and millet. Where there are boreholes, beef cattle are reared.

During their administration of the Bechuanaland Protectorate, the British built 5 Government hospitals at Francistown, Lobatse, Mahalapye, Maun and Serowe, and Mission hospitals were established independently at Kanye, Mochudi, Molepolole and Ramotswa. After Independence in 1966, the Government of Botswana built a new hospital in Gaborone and this now constitutes the central referral hospital for the country. In 1971, these 10 hospitals provided ap-

* Present address: Regional Cancer Registry, Department of Social Medicine, University of Birmingham, B15 2TJ.

This study was carried out under the auspices of the Ministry of Health, Gaborone, Botswana.

proximately 1500 beds, *i.e.* a ratio of one hospital bed per 380 people, and employed 34 medical officers, *i.e.* a ratio of one medical officer to 16,880 people.

There are no histology services within Botswana. Prior to June 1969, all specimens for histological analysis were sent to the South African Institute for Medical Research, Johannesburg, and since that date they have been sent to the Royal Army Medical College, London.

It has been pointed out by Cook and Burkitt (1970) that there are many difficulties encountered when studying the incidence of cancer in Africa, since " available medical facilities and demographic data are of a much lower standard than those usually accepted as minimal for adequate cancer registration in the developed world ". In Botswana, hospital in-patient records were the only records from which acceptable information on the patterns of cancer could be obtained. However, it is recognized that these records have still given an incomplete picture of the true incidence of cancer because: (a) many people never attended hospital, and of those who did many were treated as out-patients only, and (b) in-patient records had often been destroyed or were incomplete. Despite these limitations, the survey has been carried out using hospital in-patient records.

### METHODOLOGY

At each of the 10 hospitals in Botswana, in-patient admission registers, patients' clinical case notes and all other records such as surgical and histological records, where available, were studied to identify all cases of malignancies. The information so obtained was supplemented by the personal records of the Government Surgeon (1963–68) and by the records of the Central Laboratory, Gaborone, where, since 1967, copies of the histology records of each hospital have been retained. A total of approximately 310,000 admission register entries, 230,000 case notes and 3000 histology records were scrutinized. In order to avoid introducing bias into the survey by using a number of clerical assistants, all collection, coding and analysis of the data was done by the authors, with assistance from medical officers in the interpretation of clinical details.

The data were abstracted on an individual patient-by-patient basis and then, to eliminate multiple registration, manually sorted by name, site and related sites, hospital and date of admission. Where the records indicated that a patient had been referred to or from another hospital, a re-examination of the records for the second hospital was made to exclude from the survey cases in which cancer was subsequently disproven.

From these findings it was possible to divide the cancer patients into 3 mutually exclusive groups on the basis of the completeness and reliability of the diagnostic information available:

*Group a.*—(i) Diagnosis stated in the admission register as a particular tumour, with no further information available to substantiate this statement; (ii) diagnosis on the case notes stated as a particular tumour, but with inadequate clinical details to substantiate this statement.

*Group b.*—Diagnosis confirmed clinically.

*Group c.*—Diagnosis confirmed histologically.

Sites were classified according to the eighth revision of the International Classification of Diseases (WHO, 1965). Precancerous conditions (such as carcinoma-in-situ of the cervix uteri) were excluded. Morphological types were classified using the *Manual of Tumor Nomenclature* (American Cancer Society, 1968).

The date of admission to hospital for an illness subsequently diagnosed as cancer was taken as the starting date, since the date of admission was easier to determine than the more commonly used date of diagnosis (UICC, 1970). A preliminary inspection indicated that there were no significant seasonal variations in the number of these hospital admissions and analysis was therefore carried out only on the year of admission to hospital. The address stated was taken as the home address and was located (using *The Government Gazetter*, 1973) in one of the 5 topographical regions shown in Fig. 1. Related sub-tribes were grouped with the seven main tribes (Murdock, 1959), and Europeans were excluded from the survey.

FIG. 1.—Map of Botswana, showing location of hospitals and boundaries of topographical regions.

Description of regions

| | Region | Population | Rainfall (in.) | Topography |
|---|---|---|---|---|
| I | Ngamiland | 52,800 | 20 | Okavango Swamps cover 1/8th, remainder tree savanna with hardwood forests in NE. Malaria, trypanosomiasis endemic. Population concentrated on river banks. |
| II | Kalahari Desert | 88,400 | 12 | Shrub savanna on deep sand; no surface water. Diamond deposits and salt pans in NE. Population concentrated in NW and along banks of River Molopo; also few scattered settlements and bands of nomadic hunters (Bushmen). |
| III | North-East | 68,200 | 18 | Tree savanna with mopane. Region latticed with seasonal tributaries of River Shashe. Population scattered throughout region. |
| IV | Central | 176,200 | 18 | Tree savanna with underground water available. Rainfall lower in West than in East, where citrus fruits grown. Copper and nickel deposits in East. Population scattered throughout region. |
| V | South-East | 188,600 | 20 | Tree savanna with underground water available. Asbestos deposits at Kanye. Cattle-fattening ranches around " urban " centres of Gaborone and Lobatse. Population scattered throughout region. |

### RESULTS

From the records studied, details of 1445 patients with tumours were obtained, representing 0·46% of the hospital admissions. Although this proportion fluctuated from year to year (within the range 0·32%–0·55%), no underlying trend with time was detected.

The 10 hospitals varied in their methods of recording maternity admissions and it is estimated that the proportion of patients with tumours could be as high as 0·53% of all non-maternity admissions. These figures compare with the findings of Martin, Perry and Keen (personal communication, The Cancer Spectrum in Lesotho, 1973) in Lesotho, where the proportion of such patients among hospital admissions from 1960 to 1969 was found to be 0·50%. The statistics for Botswana and Lesotho, even allowing for some inaccuracies, are still less than half the figure of 1·28% found for cancer cases as a proportion of all admissions to Baragwanath Hospital, Johannesburg from 1951 to 1964 (Robertson, Harrington and Bradshaw, 1971). This difference is almost certainly a reflection of the lower standards of diagnostic facilities in Botswana and Lesotho, since Baragwanath is a large general hospital with patients referred from many parts of South Africa.

The quality of data collected from each hospital is shown in Table I. As can be seen, the information on the sex and address of the patient was virtually complete whereas that on age and tribe was less so, especially in the Mission hospitals. It is not thought that the missing ages are confined to any particular age group because they arise mainly from the use of the term " Adult " for most patients in 2 of the Mission hospitals. However, the marked variation in record keeping between Government and Mission hospitals is seriously reflected in an under-representation of the tribes in the areas surrounding the Mission hospitals. Although there are interesting variations in some of the frequencies by tribe, they need sub-

TABLE I.—*Summary of Data Known. Percentage of Cases for which Certain Variables were Specified at Each Hospital**

| Hospitals | No. of patients* | Sex % | Age % | Address % | Tribe % | No. of records unsubstantiated (*i.e.* Group a) |
|---|---|---|---|---|---|---|
| Government | | | | | | |
| Francistown | 148 | 99·3 | 98·6 | 88·5 | 89·2 | 91 |
| Gaborone | 114 | 100·0 | 94·7 | 97·4 | 97·4 | 19 |
| Lobatse | 74 | 97·3 | 93·2 | 93·2 | 93·2 | 83 |
| Mahalapye | 33 | 100·0 | 100·0 | 93·9 | 93·9 | 33 |
| Maun | 77 | 98·7 | 97·4 | 94·8 | 96·1 | 44 |
| Serowe | 123 | 100·0 | 99·2 | 95·9 | 94·3 | 124 |
| Sub-total | 569 | 99·3 | 97·2 | 93·7 | 93·7 | 394 |
| Mission | | | | | | |
| Kanye | 40 | 100·0 | 62·5† | 100·0 | 15·0 | 53 |
| Mochudi | 50 | 100·0 | 72·0† | 88·0 | 24·0 | 49 |
| Molepolole | 141 | 100·0 | 97·9 | 96·5 | 29·8 | 34 |
| Ramotswa | 89 | 100·0 | 98·9 | 100·0 | 9·0 | 21 |
| Sub-total | 320 | 100·0 | 89·7 | 96·6 | 21·3 | 157 |
| Total | 889‡ | 99·6 | 94·5 | 94·6 | 67·6 | 551 |

* Proven tumours only (*i.e.* Groups b and c).

† The lower percentage of cases for which age was specified at Kanye and Mochudi arose because the age was often recorded merely as " Adult ".

‡ For 5 patients the hospital was not known because their histology records, found in the Central Laboratory, Gaborone, were marked merely " Botswana " and the data could not be traced to any one particular hospital.

stantiating over a longer period of time with more complete registration and are therefore rarely discussed in this paper.

In a survey of the incidence of cancer in a developing country like Botswana, the inclusion in the analysis of clinically proven cases as well as histologically proven cases has been justified by Cook and Burkitt (1970), who showed that the bias of excluding the cases without histological proof was greater than the possible bias of including a few cases misdiagnosed on imperfect clinical evidence.

Table II gives, for the 1445 tumours found, the numbers and percentages of cases recorded by site, sex and diagnostic criterion. Of these tumours, 21·9% were clinically proven and 39·9% histologically proven. The remaining 38·2% were unsubstantiated by details of the basis of the diagnosis. The proportion of unsubstantiated diagnoses (Group a) was much higher during the first 5 years of the survey (60%) than in the following 8 years (32%), due partly to poorer diagnostic facilities and partly to the availability of fewer case notes.

It was difficult to estimate the population at risk over the period of the survey (the 1964 Census having been conducted on a *de jure* basis and the 1971 Census on a *de facto* basis). Nevertheless, minimal cancer incidence rates have been derived. Figures 2a and 2b show the age-specific incidence rate curves for all cancers in males and females respectively, compared with Bulawayo and Natal (UICC, 1970). Both figures demonstrate a flattening of the curves in the older age groups of the population in Botswana. They also indicate the degree of under-reporting of cancer in Botswana, since it is probable that the overall incidence of cancer within Southern Africa shows little regional variation. Both the under-reporting and the flattening of the age-specific incidence rate curves almost certainly reflect the poorer availability of medical services and a population (especially in the older age groups) as yet less ready to avail themselves of the facilities which do exist.

Using the Standard African Population (UICC, 1970), the age-adjusted cancer incidence rate for males in Botswana is approximately 17/100,000 and for females 19/100,000 (proven tumours only; if unsubstantiated cases are included, the rates rise to 22 and 23/100,000 respectively). These rates are lower than those reported for Baragwanath Hospital, Johannesburg (63 and 75/100,000 respectively; Robertson *et al.*, 1971) and considerably lower than Bulawayo (172 and 187/100,000; UICC, 1970) and Natal (133 and 122/100,000; UICC, 1970).

The incidence of cancer in Africa has been shown to be approximately only one-quarter of that in Western Europe and North America (UICC, 1966). It is believed therefore that the apparently very low incidence of cancer in Botswana is probably an under-representation, even though Botswana is a largely rural and agricultural society and therefore, in many respects, unlike both Bulawayo and Johannesburg. For this reason, as in other surveys based on limited data, further analysis has been carried out on a proportional frequency basis only, *i.e.* the expression of the tumours at each site as a percentage of the total number of tumours at all sites, by sex. Only the 61·8% of cases which were proven have been discussed, except where for a particular site the proportional frequency obtained when the unsubstantiated tumours were included was appreciably different from the one obtained when they were excluded.

Table III gives, for males and females, the absolute numbers of tumours of specified sites occurring in each region in Botswana and indicates where statistically significant regional differences were observed. Table IV gives, for males and females, the absolute numbers of tumours of specified sites occurring in each age group. Table V compares the patterns of frequency of tumours at specified sites in 1964–66 with those in 1970–72.

TABLE II.—*Numbers and Proportional Frequencies of Tumours Recorded at Each Site, by Sex and Diagnostic Criterion*

| | | Male | | | | | Female | | | | | |
|---|---|---|---|---|---|---|---|---|---|---|---|---|
| | Site ICD 8th Revision | Group a* | Group b* | Group c* | % b+c | % a+b+c | Group a | Group b | Group c | % b+c | % a+b+c | No. of unknown sex† |
| 140 | Lip | — | — | 1 | 0·3 | 0·2 | — | — | — | — | — | — |
| 141 | Tongue | 2 | — | 1 | 0·3 | 0·5 | — | — | 1 | 0·2 | 0·1 | — |
| 142 | Salivary gland | — | — | 2 | 0·5 | 0·3 | 2 | 1 | 3 | 0·8 | 0·8 | — |
| 143 | Gum | — | — | — | — | — | — | — | — | — | — | — |
| 144–5 | Other mouth | 1 | 3 | 6 | 2·3 | 1·5 | 1 | — | 3 | 0·6 | 0·5 | — |
| 146 | Oropharynx | — | — | — | — | — | — | — | — | — | — | — |
| 147 | Nasopharynx | 2 | — | 3 | 0·8 | 0·8 | 2 | — | 1 | 0·2 | 0·4 | — |
| 148 | Hypopharynx | — | — | — | — | — | — | — | — | — | — | — |
| 149 | Pharynx | 2 | 1 | 7 | 2·0 | 1·5 | 1 | — | 2 | 0·4 | 0·4 | — |
| 150 | Oesophagus | 25 | 20 | 22 | 10·7 | 10·3 | 9 | 1 | 1 | 0·4 | 1·4 | 2 |
| 151 | Stomach | 28 | 5 | 3 | 2·0 | 5·5 | 14 | 4 | — | 0·8 | 2·3 | — |
| 152 | Small intestine | — | — | 1 | 0·3 | 0·2 | — | — | — | — | — | — |
| 153 | Large intestine | 5 | 3 | 4 | 1·8 | 1·8 | 2 | 2 | 4 | 1·2 | 1·0 | — |
| 154 | Rectum | 2 | 3 | — | 0·8 | 0·8 | 2 | — | 2 | 0·4 | 0·5 | — |
| 155 | Liver | 74 | 20 | 14 | 8·7 | 16·5 | 24 | 5 | 6 | 2·2 | 4·5 | — |
| 156 | Gallbladder | — | — | — | — | — | — | — | 1 | 0·2 | 0·1 | — |
| 157 | Pancreas | 1 | 6 | — | 1·5 | 1·1 | 2 | 3 | — | 0·6 | 0·6 | — |
| 158 | Peritoneum | — | 2 | 1 | 0·8 | 0·5 | 3 | — | 3 | 0·6 | 0·8 | — |
| 159 | Digestive tract, unspec. | — | — | 3 | 0·8 | 0·5 | — | — | 1 | 0·2 | 0·1 | — |
| 160 | Nose and sinuses | 1 | 1 | 1 | 0·5 | 0·5 | — | — | — | — | — | — |
| 161 | Larynx | 4 | 3 | 2 | 1·3 | 1·4 | 2 | — | — | — | 0·3 | — |
| 162 | Lung, bronchus | 10 | — | 7 | 1·8 | 2·6 | 2 | 1 | 1 | 0·4 | 0·5 | — |
| 163 | Respiratory, unspec. | — | — | 1 | 0·3 | 0·2 | 1 | — | 2 | 0·4 | 0·4 | — |

| | | | | | | | | | | | | |
|---|---|---|---|---|---|---|---|---|---|---|---|---|
| 170 | Bone | 7 | 3 | 8 | 2·8 | 2·8 | 6 | 6 | 9 | 3·0 | 2·7 | — |
| 171 | Connective tissue | 10 | 1 | 21 | 5·6 | 4·9 | 6 | — | 16 | 3·2 | 2·8 | — |
| (171 | Kaposi's sarcoma) | (1) | — | (5) | (1·3) | (0·9) | (1) | — | (1) | (0·2) | (0·3) | — |
| 172 | Malignant melanoma | 4 | 4 | 15 | 4·9 | 3·5 | 6 | 4 | 14 | 3·6 | 3·1 | 1 |
| 173 | Skin | 13 | 3 | 37 | 10·2 | 8·1 | 7 | 3 | 22 | 5·1 | 4·1 | 5 |
| 174 | Breast | 1 | — | 6 | 1·5 | 1·1 | 29 | 21 | 39 | 12·1 | 11·4 | — |
| 180 | Cervix uteri | — | — | — | — | — | 80 | 99 | 98 | 39·8 | 35·4 | — |
| 181–2 | Uterus | — | — | — | — | — | 33 | 6 | 10 | 3·2 | 6·3 | — |
| 183 | Ovary | — | — | — | — | — | 7 | 3 | 9 | 2·4 | 2·4 | — |
| 184 | Other female genitalia | — | — | — | — | — | — | 3 | 15 | 3·6 | 2·3 | — |
| 185 | Prostate | 13 | 21 | 14 | 9·0 | 7·4 | — | — | — | — | — | — |
| 186 | Testis | 2 | — | 4 | 1·0 | 0·9 | — | — | — | — | — | — |
| 187·0 | Penis | 9 | 4 | 13 | 4·3 | 4·0 | — | — | — | — | — | — |
| 187·8 | Other male genitalia | 1 | — | — | — | 0·2 | — | — | — | — | — | — |
| 188 | Bladder | 5 | 5 | 4 | 2·3 | 2·1 | 5 | — | 1 | 0·2 | 0·8 | — |
| 189 | Other urinary/kidney | — | 3 | 4 | 1·8 | 1·1 | 2 | 1 | 3 | 0·8 | 0·8 | — |
| 190 | Eye | 5 | 3 | 7 | 2·6 | 2·3 | 4 | 2 | 10 | 2·4 | 2·0 | — |
| 191 | Brain | 1 | — | 1 | 0·3 | 0·3 | 1 | — | 1 | 0·2 | 0·3 | — |
| 192 | Other nervous system | — | 1 | 1 | 0·5 | 0·3 | — | — | — | — | — | — |
| 193 | Thyroid | 2 | — | — | — | 0·3 | 2 | — | 5 | 1·0 | 0·9 | — |
| 194 | Endocrine glands | — | — | 1 | 0·3 | 0·2 | — | — | 1 | 0·2 | 0·1 | — |
| 195–9 | Primary site unspec. | 25 | 6 | 17 | 5·9 | 7·4 | 19 | 9 | 19 | 5·7 | 6·0 | — |
| 200–2 | Lymphatic system | 2 | 1 | 14 | 3·8 | 2·6 | 6 | — | 3 | 0·6 | 1·1 | 1 |
| (202·0 | Burkitt's lymphoma) | — | — | (1) | (0·3) | (0·2) | (1) | — | — | — | (0·1) | — |
| 203 | Multiple myeloma | — | 1 | — | 0·3 | 0·2 | — | — | 1 | 0·2 | 0·1 | — |
| 204–7 | Leukaemia | 5 | 14 | 8 | 5·6 | 4·1 | 8 | 6 | 8 | 2·8 | 2·8 | — |
| | Total number of tumours | 262 | 137 | 254 | | | 288 | 180 | 315 | | | 9 |

* a—unsubstantiated diagnosis; b—clinical diagnosis; c—histological diagnosis. (See text for details.)

† All the tumours occurring in patients for whom sex was not recorded were proved histologically, with the exception of the malignant melanoma, the diagnosis of which was unsubstantiated.

FIG. 2a.—Age-specific incidence rates for all cancers in males, for Botswana, Bulawayo and Natal.

Reference is made to these Tables when each individual cancer site is discussed below.

(a) *Cancer of the oesophagus* (150)

The proportional frequency of cancer of the oesophagus in males in Botswana (10·7%) is moderate by African standards and falls midway in the range quoted for Southern Africa (Cook and Burkitt, 1971; Cook, 1972). It is, however, much more common than in West Africa,

FIG. 2b.—Age-specific incidence rates for all cancers in females, for Botswana, Bulawayo and Natal.

where it is still virtually unknown (Denues and Munz, 1967; Edington and Maclean, 1965). The frequency in females is much lower (0·4%) but it is increased to 1·4% by the inclusion of the unsubstantiated cases. The sex ratio therefore lies somewhere between 6 : 1 and 20 : 1 and is more like the situation observed in Johannesburg, Bulawayo or Southern Malawi than that in the Transkei, where it is common in both males and females (Cook, 1972).

TABLE III.—*Numbers of Tumours of Specified Sites Occurring in Each Region, by Sex**

| | I Ngamiland | II Kalahari Desert | III North-east | IV Central | V South-east | Unknown | Total |
|---|---|---|---|---|---|---|---|
| Males | | | | | | | |
| Oesophagus 150 | 4 | — | 10† | 6‡ | 21 | 1 | 42 |
| Liver 155 | 2 | 2 | 4 | 11 | 12 | 3 | 34 |
| Connective tissue 171 | 4 | 1 | 1 | 6 | 9 | 1 | 22 |
| Skin 172–3 | 8 | 8† | 7 | 11 | 19 | 6 | 59 |
| Prostate 185 | 3 | 2 | — | 10 | 20 | — | 35 |
| Penis 187·0 | 5† | 2 | 1 | 3 | 4 | 2 | 17 |
| All others | 16 | 5 | 14 | 61 | 74 | 12 | 182 |
| Total | 42 | 20 | 37 | 108 | 159 | 25 | 391 |
| Females | | | | | | | |
| Connective tissue 171 | — | 3 | — | 4 | 8 | 1 | 16 |
| Skin 172–3 | 5 | 8† | 3 | 10 | 13 | 4 | 43 |
| Breast 174 | 7 | 3 | 6 | 18 | 24 | 2 | 60 |
| Cervix uteri 180 | 11‡ | 12 | 11 | 53 | 105† | 5 | 197 |
| All others | 20 | 13 | 7 | 58 | 70 | 11 | 179 |
| Total | 43 | 39 | 27 | 143 | 220 | 23 | 495 |

* Proven tumours only (*i.e.* Groups b and c).
† Significantly higher than other regions combined (at the 95% level).
‡ Significantly lower than other regions combined (at the 95% level).

TABLE IV.—*Numbers of Tumours of Specified Sites Occurring in Each Age Group, by Sex**

| | Age in years | | | | | | | | |
|---|---|---|---|---|---|---|---|---|---|
| | 0–14 | 15–29 | 30–39 | 40–49 | 50–59 | 60+ | Adult | Unknown | Total |
| Males | | | | | | | | | |
| Oesophagus 150 | — | — | 3 | 12 | 13 | 13 | 1 | — | 42 |
| Liver 155 | — | 3 | 5 | 4 | 7 | 13 | 2 | — | 34 |
| Connective tissue 171 | 6 | 1 | 3 | 6 | 3 | 3 | — | — | 22 |
| Skin 172–3 | 1 | 3 | 6 | 8 | 19 | 18 | 3 | 1 | 59 |
| Prostate 185 | — | — | — | — | 4 | 28 | 3 | — | 35 |
| All others | 14 | 18 | 27 | 36 | 37 | 56 | 8 | 3 | 199 |
| Total | 21 | 25 | 44 | 66 | 83 | 131 | 17 | 4 | 391 |
| Females | | | | | | | | | |
| Connective tissue 171 | 2 | 6 | 3 | 3 | — | 2 | — | — | 16 |
| Skin 172–3 | — | 5 | 2 | 8 | 6 | 17 | 4 | 1 | 43 |
| Breast 174 | — | 2 | 11 | 17 | 17 | 11 | 2 | — | 60 |
| Cervix uteri 180 | — | 6 | 35 | 51 | 48 | 44 | 13 | — | 197 |
| All others | 24 | 27 | 23 | 24 | 38 | 39 | 4 | — | 179 |
| Total | 26 | 46 | 74 | 103 | 109 | 113 | 23 | 1 | 495 |

* Proven tumours only (*i.e.* Groups b and c).

There is some indication of geographical variation in the frequency of tumours of the oesophagus in males (see Table III). Significantly fewer tumours were found in the Central Region than in the rest of the country, and significantly more in the North-East Region. The latter region is adjacent to the south-west of Rhodesia, Francistown being only 150 miles from Bulawayo where the frequency of cancer of the oesophagus was found to be 16·2% of all male tumours (UICC, 1970). The change of frequency within Botswana is of interest in view of the dramatic gradients in frequency for this site reported from other parts of Africa and other parts of the world (Cook and Burkitt, 1971; Cook, 1972; Mahboubi *et al.*, 1973).

The frequency of cancer of the oesophagus varies widely between tribes, constituting only 2% (one out of 45 cases) of male tumours in the Bamangwato, but 56% (5 out of 9) in the Barolong. The latter figure is significantly higher than in all other tribes in Botswana.

TABLE V.—*A Comparison between* 1964–66 *and* 1970–72 *in the Proportional Frequencies of Selected Tumours**

| Site ICD 8th revision | Males 64–66 | Males 70–72 | Females 64–66 | Females 70–72 |
|---|---|---|---|---|
| Oesophagus 150 | 10·8 | 11·4 | — | 1·0 |
| Liver 155 | 10·8 | 9·6 | 4·2 | 1·5 |
| Connective tissue 171 | 9·8 | 3·0‡ | — | 3·9 |
| Skin 172–3 | 14·7 | 13·8 | 7·3 | 6·3 |
| Breast 174 | 2·0 | 1·2 | 13·5 | 12·2 |
| Cervix uteri 180 | — | — | 37·5 | 43·9 |
| Prostate 185 | 4·9 | 11·4 | — | — |
| All others | 47·1 | 49·7 | 37·5 | 31·2 |
| No. of proven tumours, all sites | 102 | 167 | 96 | 205 |
| Total no. of tumours†, all sites | 152 | 247 | 162 | 266 |

* Proven tumours only (*i.e.* Groups b and c.)

† All tumours (*i.e.* Groups a, b and c).

‡ Significantly lower in 1970–72 than in 1964–66 (at the 95% level).

There has been very little change in the pattern of frequency of cancer of the oesophagus in males with time (see Table V)—a finding which differs from those of other surveys, in particular in Baragwanath Hospital, Johannesburg (Robertson *et al.*, 1971), where the frequency of cancer of the oesophagus was shown to have doubled in males and risen five-fold in females between 1950–54 and 1960–64.

(b) *Cancer of the liver* (155)

Botswana is an extremely dry country and Oettlé (1965) has suggested that the incidence of liver cancer is usually low in populations living in dry regions. The proportional frequency of cancer of the liver in males in Botswana (8·7%) is slightly lower than the frequency observed anywhere else in Southern Africa. However, if the unsubstantiated tumours are included, the proportional frequency increases to 16·5%, which is similar to those observed in most other parts of Southern Africa. Elsewhere in the world where primary liver cancer is common, males are affected 4–5 times as frequently as females. The sex ratio in Botswana, adjusted for the population at risk, is 3·7 : 1.

The frequency of cancer of the liver in males is slightly, although not significantly, lower in Ngamiland and the South-East Region than in the other regions, between which it fluctuates little (see Table III). In females, no liver cancer was found among the 43 tumours which occurred in Ngamiland. Little was found elsewhere in the country, except in the North-East Region where, constituting 11% of the tumours (3 out of 27), it is significantly higher than in the rest of the country. Cancer of the liver has decreased in frequency only fractionally between 1964–66 and 1970–72 (see Table V), but this is not a significant change. However, Robertson *et al.* (1971) found a real decrease in its incidence and frequency between 1950–54 and 1960–64.

(c) *Tumours of connective tissue* (171)

The proportional frequencies of all tumours of connective tissue (including Kaposi's sarcoma) in both males (5·6%) and females (3·2%) are higher than reported for Bulawayo, Cape Province and Natal (UICC, 1970). In Botswana, tumours of connective tissue comprised 29% of all tumours in males under 15 years but only 8% of all tumours in females under 15 years (see Table IV). Over all ages, there has been a significant decrease in the proportional frequency of tumours of connective tissue in males from 10% in 1964–66 to 3% in 1970–72 (see Table V).

Five of the 22 proven tumours of connective tissue in males in Botswana were Kaposi's sarcoma and, in females, one out of 16. The proportional frequency of Kaposi's sarcoma in males (1·3%) is lower than in most of East, Central and Southern Africa.

(d) *Cancer of the skin* (172–3)

The frequency of tumours of the skin, including malignant melanomata, is 15·1% in males and 8·7% in females. However, Robertson *et al.* (1971) found proportional frequencies among Baragwanath Hospital in-patients in 1960–64 of only 2·0% in males and 4·0% in females. In Botswana, the proportional frequency of tumours of the skin (excluding malignant melanomata) is 10·2% in males and 5·1% in females. Nevertheless, these frequencies are still higher than those for the rest of Southern Africa, and also for most of East and Central Africa. Of these tumours, 48% occurred on the lower limbs (55% in males and 36% in females). However, it was not possible to determine the proportion of these skin tumours which originated in tropical ulcers. The frequency of tumours of the skin (including malignant melanomata) in males and females was everywhere high in Botswana, although significantly higher in the Kalahari Desert than in the rest of the country (see Table III).

(e) *Cancer of the breast—female* (174)

The proportional frequency of tumours of the breast (12·1%) is slightly higher than in other parts of Southern Africa. Although the frequency varied from 8% in the Kalahari Desert to 22% in the North-East Region (see Table III), this was not a statistically significant variation with so few observations.

(f) *Cancer of the cervix uteri* (180)

The proportional frequency of tumours of the cervix uteri was 39·8%. It was significantly lower in Ngamiland than in the rest of Botswana and significantly higher in the South-East Region (the most densely populated region), being 48% of all female tumours in the latter region (see Table III). This level is higher than in all surveys quoted by Cook and Burkitt (1971), with the exception of one Johannesburg survey (Robertson, 1969), and is comparable with the frequency of 54% found in Lesotho by Martin *et al.* (personal communication, 1973). Robertson *et al.* (1971) found that the Batswana* women had the highest rate of cancer of the cervix uteri per 1000 hospital admissions of all Southern African tribes.

(g) *Cancer of the prostate* (185)

The proportional frequency of tumours of the prostate (9·0%) is higher than those reported for Bulawayo, Cape Province and Natal (UICC, 1970). Within Botswana, the number of tumours of the prostate was significantly lower in the North-East Region than in the South-East Region, but in neither region was the number of tumours significantly different from the total for the rest of the country (see Table III). Although there was an increase in the proportional frequency of cancer of the prostate from 5% in 1964–66 to 11% in 1970–72 (see Table V), it was not statistically significant. Cancer of the prostate was overwhelmingly the most common tumour in males over 60 years of age, and the data support the findings of Cook, Doll and Fellingham (1969) that cancer of the prostate occurs later in life and shows a sharper increase with age than almost any other tumour.

(h) *Cancer of the penis* (187·0)

Tumours of the penis are more frequent in Botswana (4·3%) than in the rest of Southern Africa, but less frequent than in parts of East and Central Africa. The area of highest proportional frequency was Ngamiland where, at 12%, the frequency was significantly higher than in the rest of the country (see Table III). It is of interest that Robertson *et al.*

* Botswana is the country, the Batswana are the people.

(1971) found that the Batswana men had amongst the highest rates of cancer of the penis per 1000 hospital admissions of Southern African tribes studied, and that less than 20% of the Batswana men practised circumcision.

We should like to thank the Board of Control: Deferred Pay Interest Fund of the Mine Labour Organization of South Africa for their grant which enabled this survey to be carried out. For the initial guidance, we appreciate the encouragement from Dr P. Keen of the Cancer Research Unit, South African Institute for Medical Research, Johannesburg, and from Dr P. Martin of the University of Botswana, Lesotho and Swaziland, Roma, Lesotho. We should like to thank the Government of Botswana for supporting the project and for permitting us unrestricted access to all available medical records. However, this work would not have been possible without the full co-operation of the medical, nursing and laboratory staff throughout Botswana, to whom we wish to express our grateful thanks for all advice, assistance and hospitality received. In particular, we wish to acknowledge the assistance received from Dr I. Kennedy, Government Surgeon, 1963–68 and Dr A. Merriweather, Superintendent, Scottish Livingstone Hospital, Molepolole since 1945. For assistance with the analysis of the data, we are grateful to Professor P. D. Griffiths and the computer staff of the department of Clinical Chemistry, Ninewells Hospital, Dundee, Scotland. Finally, we should like to thank Miss Paula Cook and Dr J. A. H. Waterhouse for their constructive criticisms of the draft of this paper.

### REFERENCES

American Cancer Society (1968) *Manual of Tumor Nomenclature and Coding.* American Cancer Society Inc.

Central Statistics Office (1971) *Report on the Population Census.* Gaborone, Botswana: Ministry of Finance and Development Planning.

Central Statistics Office (1973) *Guide to the Villages of Botswana.* Gaborone, Botswana: Ministry of Finance and Development Planning.

Cook, P. (1972) *Cancer of the Oesophagus in Africa.* London: Medical Research Council.

Cook, P. & Burkitt, D. (1970) *An Epidemiological Study of Seven Malignant Tumours in East Africa.* London: Medical Research Council.

Cook, P. & Burkitt, D. (1971) Cancer in Africa. *Br. med. Bull.*, **27**, 14.

Cook, P., Doll, R. & Fellingham, S. (1969) A Mathematical Model for the Age Distribution of Cancer in Man. *Int. J. Cancer*, **4**, 93.

Denues, A. R. T. & Munz, W. (1967) Malignancies at the Hospital of Dr Albert Schweitzer, Lambarene, Gabon, 1950–55. *Int. J. Cancer*, **2**, 406.

Edington, G. M. & Maclean, C. M. U. (1965) A Cancer Rate Survey in Ibadan, Western Nigeria, 1960–63. *Br. J. Cancer*, **19**, 471.

Mahboubi, E., Kmet, J., Cook, P. J., Day, N. E., Ghadirian, P. & Salmasizadeh, S. (1973) Oesophageal Cancer Studies in the Caspian Littoral of Iran: The Caspian Cancer Registry. *Br. J. Cancer*, **28**, 197.

Murdock, G. (1959) *Africa: Its People and Their Culture History.* New York: McGraw-Hill.

Oettlé, A. (1965) The Aetiology of Primary Carcinoma of the Liver in Africa. *S. Afr. med. J.*, **39**, 817.

Robertson, M. (1969) Clinical Observations on Cancer Patterns at the Non-White Hospital, Baragwanath, Johannesburg, 1948–1964. *S. Afr. med. J.*, **43**, 915.

Robertson, M., Harrington, J. & Bradshaw, E. (1971) The Cancer Pattern in Africans at Baragwanath Hospital, Johannesburg. *Br. J. Cancer*, **25**, 377.

UICC (1966) *Cancer Incidence in Five Continents*, 1. Ed. R. Doll, P. Payne & J. Waterhouse. Berlin, Heidelberg, New York: Springer-Verlag.

UICC (1970) *Cancer Incidence in Five Continents*, 2. Ed. R. Doll, C. Muir & J. Waterhouse. Berlin, Heidelberg, New York: Springer-Verlag.

World Health Organization (1965) *Manual of International Statistical Classification of Diseases, Injuries and Causes of Death, Vol. I*, Geneva.